# Effect of Renal Impairment on Clinical Outcomes After Mitral Valve Transcatheter Edge-to-Edge Repair

**DOI:** 10.1016/j.jacasi.2024.10.025

**Published:** 2025-01-14

**Authors:** Kazuki Tanaka, Junichi Yamaguchi, Masafumi Yoshikawa, Eiji Shibahashi, Hisao Otsuki, Takanori Kawamoto, Chihiro Koyanagi, Yusuke Inagaki, Tomohito Kogure, Masanori Yamamoto, Mike Saji, Masahiko Asami, Masaki Nakashima, Yusuke Enta, Shinichi Shirai, Masaki Izumo, Shingo Mizuno, Yusuke Watanabe, Makoto Amaki, Kazuhisa Kodama, Shunsuke Kubo, Yoshifumi Nakajima, Toru Naganuma, Hiroki Bota, Yohei Ohno, Masahiro Yamawaki, Hiroshi Ueno, Kazuki Mizutani, Toshiaki Otsuka, Kentaro Hayashida, OCEAN-Mitral Investigators

**Affiliations:** a Department of Cardiology Tokyo Woman’s Medical University, Tokyo, Japan; b Department of Cardiology, Toyohashi Heart Center, Toyohashi, Japan; c Department of Cardiology, Nagoya Heart Center, Nagoya, Japan; d Department of Cardiology, Gifu Heart Center, Gifu, Japan; e Department of Cardiology, Sakakibara Heart Institute, Okayama, Japan; f Division of Cardiovascular Medicine, Department of Internal Medicine, Toho University Faculty of Medicine, Tokyo, Japan; g Division of Cardiology, Mitsui Memorial Hospital, Tokyo, Japan; h Department of Cardiology, Sendai Kosei Hospital, Sendai, Japan; i Division of Cardiology, Kokura Memorial Hospital, Kitakyushu, Japan; j Division of Cardiology, St. Marianna University School of Medicine Hospital, Kawasaki, Japan; k Department of Cardiology, Shonan Kamakura General Hospital, Kanagawa, Japan; l Department of Cardiology, Teikyo University School of Medicine, Tokyo, Japan; m Department of Cardiology, National Cerebral and Cardiovascular Center, Suita, Japan; n Division of Cardiology, Saiseikai Kumamoto Hospital Cardiovascular Center, Kumamoto, Japan; o Department of Cardiology, Kurashiki Central Hospital, Kurashiki, Japan; p Division of Cardiology, Department of Internal Medicine, Iwate Medical University, Iwate, Japan; q Department of Cardiology, New Tokyo Hospital, Chiba, Japan; r Department of Cardiology, Sapporo Higashi Tokushukai Hospital, Sapporo, Japan; s Department of Cardiology, Tokai University School of Medicine, Isehara, Japan; t Saiseikai Yokohama City Eastern Hospital, Kanagawa, Japan; u Second Department of Internal Medicine, Toyama University Hospital, Toyama, Japan; v Division of Cardiology, Department of Medicine, Kinki University Faculty of Medicine, Osaka, Japan; w Department of Hygiene and Public Health, Nippon Medical School, Tokyo, Japan; x Department of Cardiology, Keio University School of Medicine, Tokyo, Japan

**Keywords:** heart failure, major adverse cardiovascular event(s), mitral regurgitation, mitral valve transcatheter edge-to-edge repair, renal impairment

## Abstract

**Background:**

Renal impairment is associated with poor clinical outcomes in patients with cardiovascular diseases. Some studies have revealed the impact of renal impairment on the clinical outcomes of patients who underwent mitral valve transcatheter edge-to-edge repair (M-TEER). However, limited data are available regarding the impact of baseline renal impairment after M-TEER in Asian-Pacific patients with heart failure and severe mitral regurgitation.

**Objectives:**

This study sought to examine the effect of renal impairment on clinical outcomes after M-TEER using a large-scale nationwide registry in Japan.

**Methods:**

A total of 2,150 patients enrolled in the OCEAN-Mitral (Optimized Catheter Valvular Intervention) registry were divided into 3 groups according to the estimated glomerular filtration rate (eGFR) before M-TEER: normal eGFR (≥60 mL/min/1.73 m^2^) (n = 291), renal impairment (<60 mL/min/1.73 m^2^) (n = 1,746), and dialysis (n = 113). The impact of renal impairment and dialysis on major adverse cardiovascular events (MACE) (a composite of all-cause death and hospitalization for heart failure) was examined.

**Results:**

Kaplan-Meier analysis revealed that the renal impairment and dialysis groups had a significantly higher incidence of MACE (survival rates at 2 years: normal eGFR, 74.2% [95% CI: 66.9%-80.1%] vs renal impairment, 63.9% [95% CI: 61.0%-66.6%] vs dialysis, 50.9% [95% CI: 38.2%-62.2%]; *P <* 0.001). Multivariate Cox regression analysis identified dialysis as the strongest independent predictor of MACE (HR: 1.95; 95% CI: 1.33-2.85; *P* < 0.001).

**Conclusions:**

Renal impairment was associated with an increased incidence of major adverse events, and dialysis was the strongest independent predictor of poor clinical outcomes after M-TEER in Asian-Pacific patients.

Mitral regurgitation (MR) is a common valvular heart disease in developed countries.^1^, ^2^, ^3^ Mitral valve surgery is the treatment of choice for patients with severe primary and secondary MR.^4^^,^^5^ However, a significant proportion of patients with severe MR have a high surgical risk, with multiple comorbidities precluding them from undergoing open mitral valve surgery.^6^ Mitral valve transcatheter edge-to-edge repair (M-TEER) using the MitraClip (Abbott Vascular) is an established treatment option with increasing clinical application in patients with high surgical risk.^7^, ^8^, ^9^ Some previous studies have revealed that one of the significant comorbidities that often contributes to high surgical risk in patients with severe MR is baseline renal impairment,^10^, ^11^, ^12^, ^13^ which adversely affects the outcomes of M-TEER procedures and mitral valve surgery.^14^^,^^15^ However, limited studies have focused on the impact of baseline renal impairment on clinical outcomes after M-TEER in Asian-Pacific patients. Moreover, because M-TEER is a procedure for high-risk surgical patients with various comorbidities with expected higher mortality in general, predicting clinical outcomes after the procedure is crucial. This study aimed to evaluate the effect of renal impairment on clinical outcomes after M-TEER in Asian-Pacific patients using a nationwide registry in Japan.

## Methods

### Study design and population

This is a retrospective analysis of the OCEAN (Optimized transCathEter vAlvular intervention) transcatheter mitral valve intervention (OCEAN-Mitral) registry, which is an ongoing, prospective, investigator-initiated, multicenter registry used to assess the safety and efficacy of M-TEER in patients with significant MR. A total of 21 Japanese institutions participated in the registry. Between April 2018 and June 2021, 2,150 consecutive symptomatic patients with MR underwent M-TEER.^16^^,^^17^ Baseline data, including patient comorbidities, echocardiographic findings, and procedural details, were collected. Echocardiographic assessments of MR severity and M-TEER procedure were performed at the discretion of the interdisciplinary heart team at each participating hospital.

**Figure 1:**
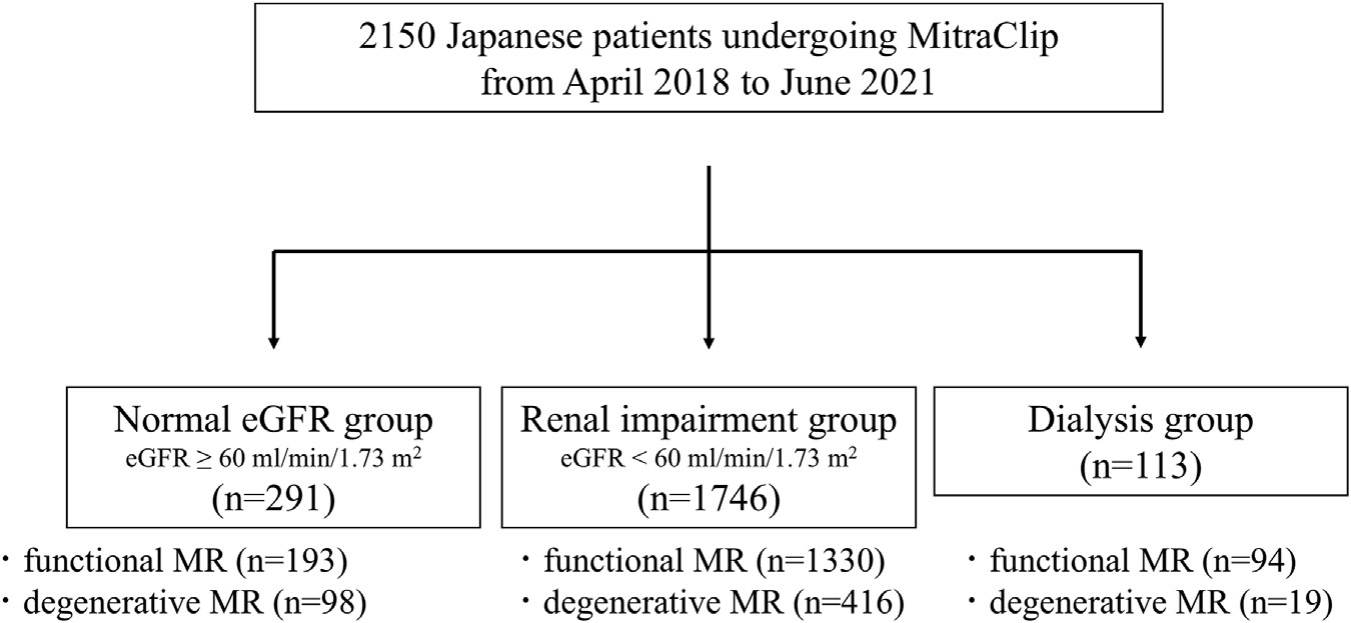
Patient’s Flow The 2,150 patients undergoing mitral valve transcatheter edge-to-edge repair using MitraClip were categorized into 3 groups according to their renal function as follows: normal estimated glomerular filtration rate (eGFR) group: n = 291, 13.5%; renal impairment group: n = 1,746, 81.2%; and dialysis group: n = 113, 5.3%. The etiology of mitral regurgitation (MR) in each group was also noted.

The enrolled patients were divided into 3 groups according to the estimated glomerular filtration rate (eGFR) calculated using the Modification of Diet in Renal Disease (MDRD) equation^18^ before TEER: normal eGFR (≥60 mL/min/1.73 m^2^) (n = 291), renal impairment (<60 mL/min/1.73 m^2^) (n = 1,746), and dialysis groups (n = 113) (Figure 1). The clinical outcomes of the 3 groups were evaluated.

Because the etiology of MR may affect the clinical outcomes, we additionally performed the same analysis on functional MR (n = 1,617) and degenerative MR (n = 533).

### Study endpoints

The primary endpoint of this study was major adverse cardiovascular events (MACE) (a composite of all-cause death and heart failure [HF] hospitalization). The secondary endpoints were the components of the primary endpoint. Survival status was determined through outpatient visits and telephone interviews with patients or relatives.

### Statistical analysis

Continuous variables were expressed as mean ± SD or median (Q1-Q3) depending on the distribution of data, whereas categorical variables were expressed as frequencies and percentages. Comparisons were made using the Student's *t*-test for normally distributed continuous variables or the Mann-Whitney *U* test for asymmetrically distributed continuous variables. Categorical variables were compared using the chi-square test or Fisher exact test. We performed missing data analysis and visualized it as Supplemental Figure 1. The Kaplan-Meier test was used to estimate the incidence of MACE, all-cause death, and HF hospitalization after M-TEER, and the differences in event-free rates among the 3 groups were compared using a log-rank test. The survival rates of specific time points of each event were estimated from the Kaplan-Meier analysis. Univariate and multivariate analyses were performed to identify independent predictors of the primary endpoint. After univariate analysis, multivariate Cox hazard regression analyses were performed using the covariates that were considered potential confounding factors with the clinical events of interest from previous reports (age, sex, body mass index, diabetes, atrial fibrillation, prior stroke, chronic obstructive pulmonary disease, smoking, NYHA functional class, left ventricular end-diastolic diameter, left ventricular end-systolic diameter, etiology of MR, and postprocedural MR).^19^^,^^20^ The results of the multiple variable Cox analyses were reported as HRs with 95% CIs and *P* values. The proportional hazard assumption was assessed and satisfied graphically by plotting log (−log) survival curves against log survival time for each group and verifying that the curves were parallel. The relative treatment effect across the eGFR groups between functional MR and degenerative MR) was analyzed using a Cox hazard model. Statistical significance was set at *P <* 0.050. All statistical analyses were performed by an independent physician using the statistical analysis software JMP Pro 17, version 17.0.0 (SAS Institute) and R version 4.2.1 (R Foundation).

### Ethical considerations

The ethics committee of each participating hospital approved the study protocol. All patients provided written informed consent before participating in the study. Patient enrollment and the study were conducted in accordance with the principles of the Declaration of Helsinki. This study was registered with the University Hospital Medical Information Network Clinical Trials Registry and approved by the International Committee of Medical Journal Editors (UMIN000023653).

## Results

### Baseline clinical characteristics

Of the 2,150 patients who underwent M-TEER, only 14% (n = 291) had eGFR ≥60 mL/min/1.73 m^2^, whereas 81% (n = 1,746) had eGFR <60 mL/min/1.73 m^2^, and 5% (n = 117) were on dialysis. Regarding baseline characteristics, there were significant differences among the 3 groups in terms of age, sex, the Society of Thoracic Surgeons (STS) score for mitral valve replacement, smoking, history of cardiovascular disease, and medication at discharge. As renal impairment worsened, patients aged and showed many cardiovascular comorbidities, resulting in higher STS scores in patients with renal impairment and those on dialysis (Table 1).

**Table 1:** Baseline Characteristics

	Normal eGFR Group (n = 291)	Renal Impairment Group (n = 1,746)	Dialysis Group (n = 113)	*P* Value
Age, y	74.1 ± 12.1	79.3 ± 8.8	74.7 ± 9.8	<0.001
Male	185 (63.6)	945 (54.1)	79 (69.9)	<0.001
BMI, kg/m^2^	21.2 ± 3.9	21.3 ± 3.5	21.0 ± 3.4	0.51
NYHA functional class	2.7 ± 0.8	2.6 ± 0.7	2.8 ± 0.8	0.58
Frailty score ≥7	22 (7.7)	82 (4.9)	4 (3.7)	0.098
STS score MV replacement	7.5 ± 5.3	11.3 ± 8.2	18.0 ± 10.6	<0.001
EuroScore II	4.1 ± 4.5	7.3 ± 6.7	7.5 ± 6.4	<0.001
Hypertension	184 (63.2)	1,190 (68.2)	78 (69.0)	0.24
Diabetes	52 (17.9)	377 (21.6)	26 (23.0)	0.31
Current smoking	25 (8.6)	107 (6.1)	13 (11.5)	0.035
Atrial fibrillation/flutter	152 (52.2)	1,162 (66.6)	54 (47.8)	<0.001
CRTP/D	20 (6.9)	195 (11.2)	7 (6.2)	0.028
Prior coronary artery disease	73 (25.1)	634 (36.3)	67 (59.3)	<0.001
Old myocardial infarction	38 (13.1)	421 (24.1)	43 (38.1)	<0.001
Prior PCI/CABG	67 (23.0)	574 (32.9)	60 (53.1)	<0.001
Peripheral artery disease	16 (5.5)	178 (10.2)	30 (26.5)	<0.001
Prior stroke	32 (11.0)	199 (11.4)	16 (14.2)	0.65
COPD	35 (12.0)	171 (9.8)	9 (8.0)	0.38
Medication at discharge				
Dual antiplatelet therapy	43 (14.8)	190 (10.9)	35 (31.0)	<0.001
Oral anticoagulation therapy	176 (60.7)	1,233 (71.1)	34 (30.4)	<0.001
ACEI/ARB	199 (68.9)	1,121 (64.6)	46 (41.1)	<0.001
MRA	188 (64.8)	1,030 (59.4)	14 (12.5)	<0.001
Echocardiographic parameters				
Functional MR	193 (66.3)	1,330 (76.2)	94 (83.2)	<0.001
LV end-diastolic diameter, mm	57.7 ± 11.0	57.0 ± 10.2	59.0 ± 8.4	0.11
LV end-systolic diameter, mm	44.2 ± 14.2	44.1 ± 13.2	48.1 ± 11.5	0.007
LV ejection fraction, %	46.0 ± 17.8	45.5 ± 16.1	40.1 ± 15.0	0.002
LA volume, mL	134.0 ± 75.2	139.1 ± 90.0	119.9 ± 49.3	0.068
EROA, cm^2^	0.4 ± 0.4	0.4 ± 0.2	0.4 ± 0.4	0.012
Regurgitant volume of MR, mL	58.3 ± 27.5	56.1 ± 25.9	57.1 ± 24.5	0.38

Values are mean ± SD or n (%).

ACEI = angiotensin-converting enzyme inhibitor; ARB = angiotensin receptor blocker; BMI = body mass index; CABG = coronary artery bypass grafting; COPD = chronic obstructive pulmonary disease; CRTP/D = cardiac resynchronization therapy pacemaker/defibrillator; EROA = effective regurgitant orifice area; LA = left atrium; LV = left ventricle; MR = mitral regurgitation; MRA = mineralocorticoid receptor antagonist; MV = mitral valve; PCI = percutaneous coronary intervention; STS = Society of Thoracic Surgeons.

### Clinical outcomes

The median follow-up period was 436 days (Q1-Q3: 363-733 days). Among the 2,150 enrolled patients, 607 MACE, 366 all-cause deaths, and 371 hospitalizations caused by HF occurred at 2 years after M-TEER. The details of all-cause deaths are listed in Supplemental Table 1.

**Figure 2:**
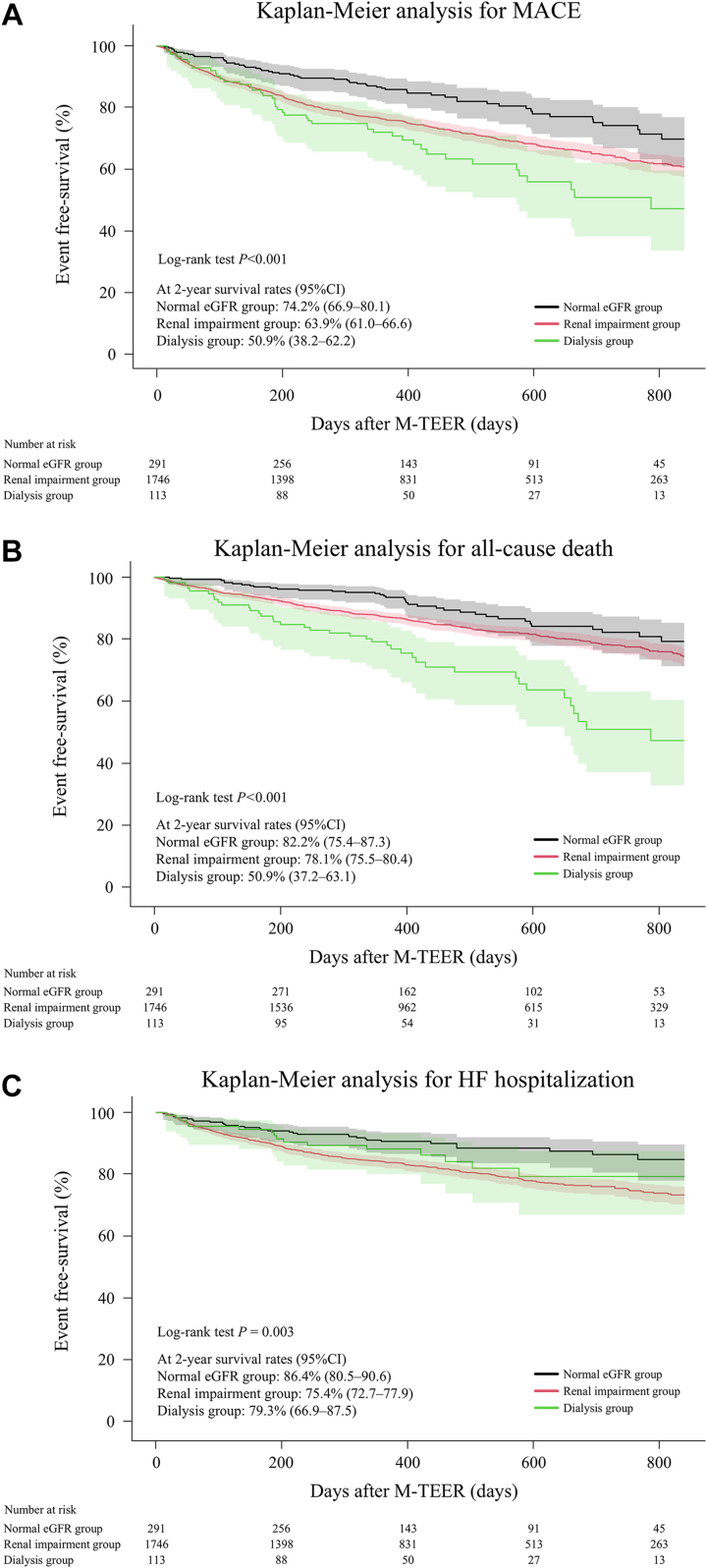
Kaplan-Meier Analysis for the Endpoints Kaplan-Meier analysis revealed that patients with renal impairment and those on dialysis had a significantly higher incidence of major adverse cardiovascular event (MACE) (A), all-cause death (B), and heart failure (HF) hospitalization (C) than those with normal estimated glomerular filtration rate (eGFR). M-TEER = mitral valve transcatheter edge-to-edge repair.

Kaplan-Meier analysis revealed that patients with renal impairment and on dialysis had a significantly higher incidence of MACE (survival rates at 2 years: normal eGFR, 74.2% [95% CI: 66.97%-80.1%] vs renal impairment, 63.9% [95% CI: 61.0%-66.6%] vs dialysis, 50.9% [95% CI: 38.2%-62.2%]; *P <* 0.0001), all-cause death (survival rates at 2 years: 82.2% [95% CI: 75.4%-87.3%] vs 78.1% [95% CI: 75.5%-80.4%] vs 50.9% [95% CI: 37.2%-63.1%]; *P* < 0.001), and HF hospitalization (survival rates at 2 years: 86.4% [95% CI: 80.5%-90.6%] vs 75.4% [95% CI: 72.7%-77.9%] vs 79.3% [95% CI: 66.9%-87.5%]; *P =* 0.003) (Figures 2A to 2C)

### Comparison of functional and degenerative MR

**Figure 3:**
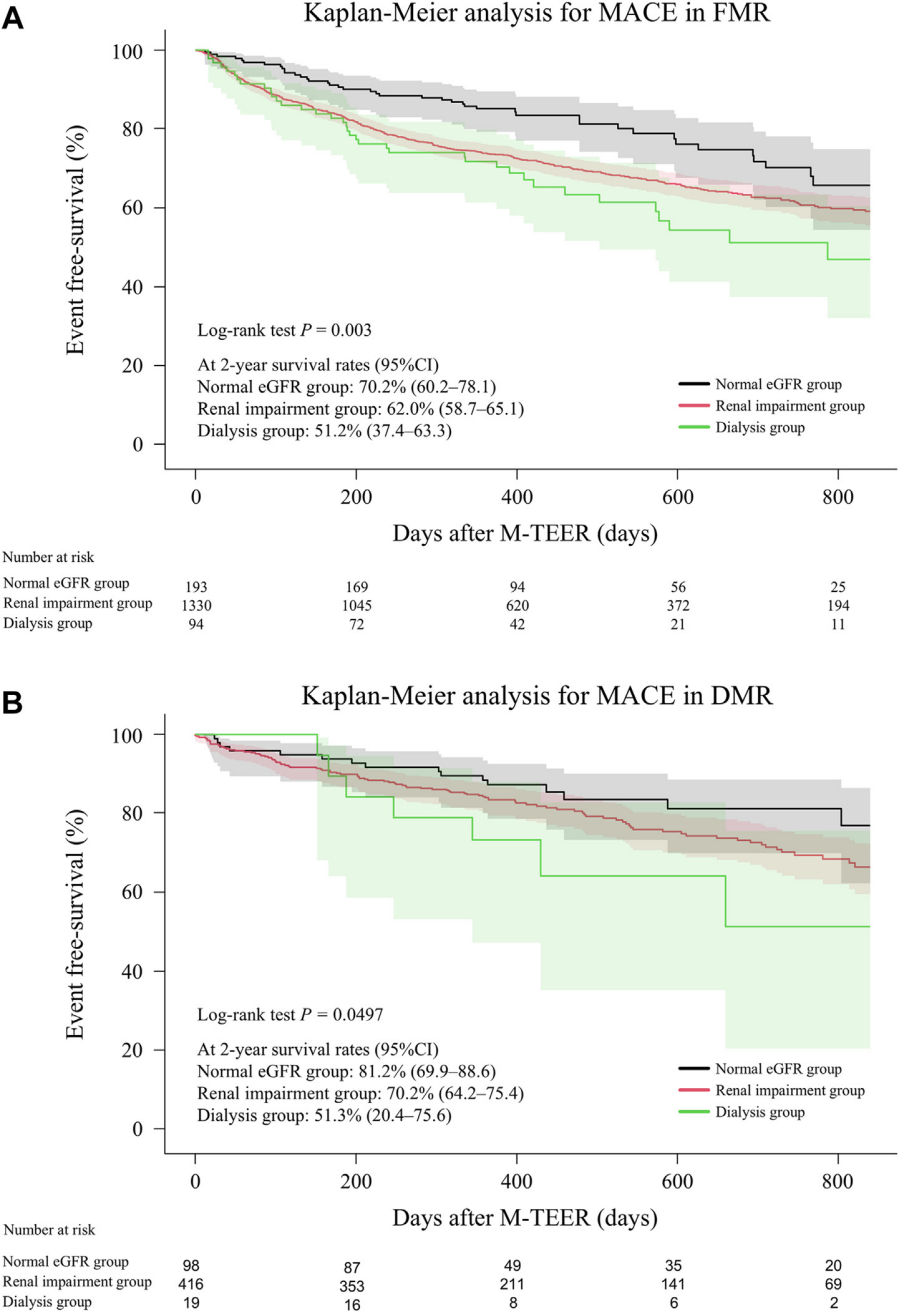
Kaplan-Meier Analysis for MACE in FMR and DMR Comparison of functional mitral regurgitation (FMR) and degenerative mitral regurgitation (DMR). Patients on dialysis showed a higher incidence of MACE than those with normal eGFR and renal impairment in the FMR (A) and DMR (B) groups. Abbreviations as in Figure 2.

The same trend was observed in the subgroup analysis of functional MR (n = 1,617) and degenerative MR (n = 533). Patients on dialysis showed a higher incidence of MACE than those with normal eGFR and renal impairment in the functional and degenerative MR groups (survival rates at 2 years: normal eGFR, 70.2% [95% CI: 60.2%-78.1%] vs renal impairment, 62.0% [95% CI: 58.7%-65.1%] vs dialysis, 51.2% [95% CI: 37.4%-63.3%]; log-rank *P =* 0.003 in the functional MR group and 81.2% [95% CI: 69.9%-88.6%] vs 70.2% [95% CI: 64.2%-75.4%] vs 51.3% [95% CI: 20.4%-75.6%]; log-rank *P =* 0.050 in the degenerative MR group) (Figures 3A and 3B). The same trends were observed in the incidence of all-cause death in the functional and degenerative MR groups as follows: survival rates at 2 years: normal eGFR, 80.5% [95% CI: 71.2%-87.1%] vs renal impairment, 77.6% [95% CI: 74.7%-80.3%] vs dialysis, 50.9% [95% CI: 35.5%-64.4%]; log-rank *P <* 0.001 in the functional MR group and 84.9% [95% CI: 73.5%-91.6%] vs 79.7% [95% CI: 74.2%-84.2%] vs 51.3% [95% CI: 20.4%-75.6%]; log-rank *P =* 0.003 in the degenerative MR group) (Supplemental Figures 2A and 2B). Regarding HF hospitalization, patients with renal impairment and those on dialysis showed a significantly higher incidence in the functional MR group but not in the degenerative MR group as follows (survival rates at 2 years: normal eGFR, 83.6% [95% CI: 75.4%-89.3%] vs renal impairment, 72.3% [95% CI: 69.1%-75.2%] vs dialysis, 75.4% [95% CI: 61.3%-84.9%]; log-rank *P =* 0.013 in the functional MR group and 91.5% [95% CI: 82.4%-95.9%] vs 85.3% [95% CI: 80.2%-89.2%] vs 100.0%; log-rank *P =* 0.240 in the degenerative MR group) (Supplemental Figures 2C and 2D).

The interaction analysis using Cox regression revealed no significant interaction between functional and degenerative MR across eGFR categories for each outcome: MACE (*P* for interaction = 0.61), all-cause death (*P* for interaction = 0.82), and HF hospitalization (*P* for interaction = 0.57).

### Predictors of MACE

In the multivariate analysis, the adjusted risks of renal impairment and dialysis relative to normal eGFR for MACE remained significant (adjusted HR: 1.50 [95% CI: 1.14-1.96]; *P =* 0.034 and adjusted HR: 1.95 [95% CI: 1.33-2.85]; *P <* 0.001, respectively). Other independent predictors of MACE are presented in Table 2.

**Table 2:** Cox Regression Analysis for MACE (All-Cause Death + HF Hospitalization)

	HR (95% CI)	*P* Value
Baseline renal function		
Normal eGFR	Reference	
Renal impairment	1.50 (1.14-1.96)	0.003
Dialysis	1.95 (1.33-2.85)	<0.001
Age	1.02 (1.01-1.03)	<0.001
Male	1.23 (1.02-1.48)	0.03
BMI	0.95 (0.93-0.98)	<0.001
Atrial fibrillation/flutter	1.26 (1.06-1.49)	0.008
NYHA functional class	1.55 (1.39-1.73)	<0.001
LVDs	1.02 (1.01-1.04)	0.004
Postprocedural MR (>moderate)	1.25 (1.03-1.53)	0.027

eGFR = estimated glomerular filtration rate; LVDs = left ventricular end-systolic diameter; other abbreviations as in Table 1.

Regarding the secondary endpoints, both renal impairment and dialysis were independent predictors for all-cause death (adjusted HR: 1.46 [95% CI: 1.04-2.07]; *P =* 0.031 and adjusted HR: 2.87 [95% CI: 1.84-4.47]; *P <* 0.001, respectively). However, renal impairment was an independent predictor for HF hospitalization (adjusted HR: 1.68 [95% CI: 1.18-2.42]; *P =* 0.005), but not dialysis (adjusted HR: 1.20 [95% CI: 0.66-2.18]; *P =* 0.55).

## Discussion

The primary findings of this study are as follows: 1) the baseline renal impairment was pervasive in patients undergoing M-TEER, with a high prevalence among patients on dialysis; 2) patients with renal impairment and undergoing dialysis showed a significantly higher incidence of MACE (Central Illustration); 3) patients undergoing dialysis showed a significantly higher incidence of MACE in the functional and degenerative MR groups; and 4) dialysis was the strongest independent predictor of MACE.

**Central Illustration:**
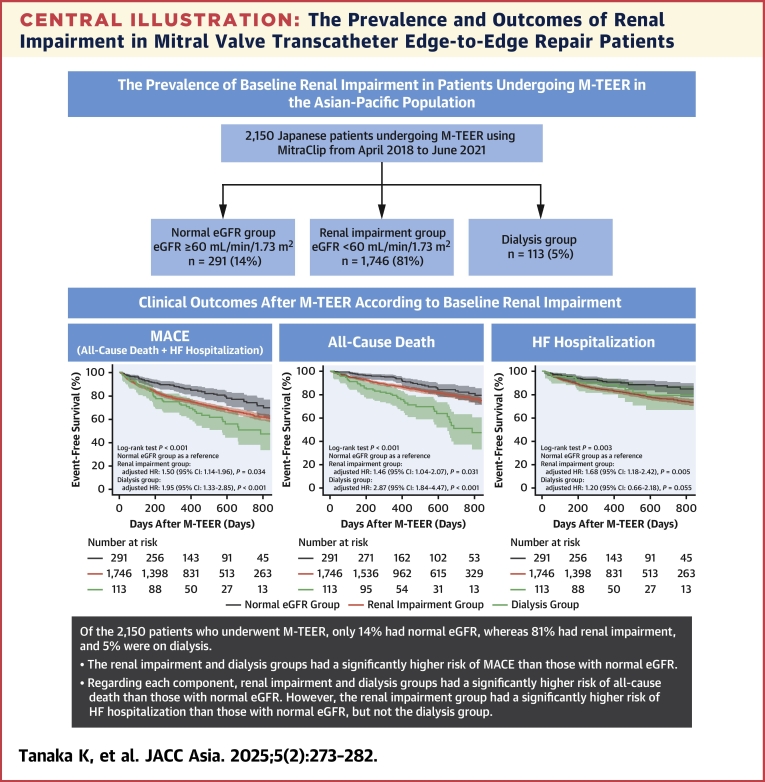
The Prevalence and Outcomes of Renal Impairment in Mitral Valve Transcatheter Edge-to-Edge Repair Patients Baseline renal impairment was associated with an increased incidence of major adverse cardiovascular events (MACE) after mitral valve transcatheter edge-to-edge repair (M-TEER) for severe mitral regurgitation. Dialysis was the strongest predictor of MACE. eGFR = estimated glomerular filtration rate; HF = heart failure.

M-TEER using MitraClip is an established treatment in high-risk surgical candidates with severe MR.^21^ In a real-world Japanese setting of M-TEER, the prevalence of preoperative renal impairment, including dialysis, was approximately 85%, which was higher than that in previous studies from Western countries.^19^^,^^22^ Nonetheless, Kubo et al^16^ reported favorable clinical outcomes; mortality and HF hospitalization rates after 1 year were 12.3% and 15%, respectively, in the same cohort as in the present study. The mortality rate was similar to those of previous reports as follows: the Japanese postmarketing study (14.9%), the device arm of the COAPT trial (18.8%), and the MitraClip EXPAND study (14.9%).^9^^,^^23^^,^^24^ Moreover, it was numerically lower than those in the independent German TRAMI (Transcatheter Mitral Valve Interventions) registry (20.2%) and the TVT (Transcatheter Valve Therapy) registry (25.8%), which included fewer patients with renal impairment.^25^^,^^26^ It has been suggested that accumulated experience with M-TEER has enabled the safe and effective treatment of higher-risk patients.

Several studies have evaluated the impact of baseline renal impairment on clinical outcomes after M-TEER; however, the data are limited. The initial EVEREST (Endovascular Valve Edge-to-Edge REpair Study) registry of the M-TEER using MitraClip excluded patients with renal disease, and only 3.3% of the patients of EVEREST II had renal disease.^7^^,^^27^ A recent study by Sisinni et al^22^ evaluated the impact of chronic kidney disease on patients with HF and functional MR who underwent M-TEER. At the 5-year Kaplan-Meier analysis, the primary clinical endpoint, including a composite of overall death and first rehospitalization for HF, occurred in 60% of patients with normal eGFR, 73% of patients with mild-to-moderate chronic kidney disease, and 91% of patients with severe chronic kidney disease. Our results, using data from an Asian-Pacific cohort, were consistent with those showing worse outcomes for renal impairment after M-TEER. In addition, we observed deteriorating clinical outcomes in patients undergoing dialysis.

Among the details of all-cause deaths, infection appeared to be one of the leading causes of noncardiac death (Supplemental Table 1). Previous studies have established that infection is a common cause of death and hospitalization in patients with HF,^28^, ^29^, ^30^ and patients with renal impairment and those undergoing dialysis are at high risk of infections. Accordingly, a preventive strategy against infection in patients with renal impairment and those on dialysis may be a crucial treatment target after M-TEER.

Impaired renal function is a poor prognostic factor for various cardiovascular procedures.^12^^,^^18^^,^^31^ It also decreases serum phosphate excretion. Increased phosphate combines with calcium to promote arteriosclerosis, eventually increasing the incidence of cardiovascular events.^32^ In particular, patients undergoing dialysis have a higher frequency of advanced atherosclerosis and comorbidities than those without chronic kidney disease, resulting in significantly higher rates of cardiovascular events. Our study showed that dialysis was the strongest independent predictor of mortality after M-TEER, which is consistent with the results of a previous report.^33^ This could be attributed to the general high-risk state and higher bleeding risk in patients on dialysis, which are associated with comorbidities. Notably, the incidences of HF in the dialysis and renal impairment groups were similar. Patients undergoing dialysis have their fluid levels checked at the clinic almost every 2 days, which could have resulted in fewer HF hospitalizations.

Finally, in a separate examination of patients with functional and degenerative MR, our study revealed a similar tendency in cardiovascular event occurrence regardless of renal impairment. These results are consistent with those of a previous meta-analysis, which showed that M-TEER is a good option for high-risk patients with both functional and degenerative MR.^34^

### Study limitations

First, this was a retrospective analysis of data from a prospective multicenter registry. Thus, the unbalanced number of participants in each group may have resulted in biased sampling, and there is a possibility of confounding and residual bias exists. The results of this study may generate hypotheses and should be interpreted cautiously. Second, we could not evaluate the effects of worsening renal function during follow-up. Third, the severity of each comorbidity was not assessed. Finally, echocardiographic parameters were not evaluated in an independent core laboratory.

## Conclusions

Baseline renal impairment was associated with an increased incidence of MACE after M-TEER for severe MR, and dialysis was the strongest independent predictor of poor clinical outcomes in Asian-Pacific patients. With the expanding use of M-TEER in clinical practice, tailor-made treatment strategies for these high-risk subsets are warranted in real-world settings.

## Funding Support and Author Disclosures

The OCEAN-Mitral registry, which is part of the OCEAN-SHD registry, was supported by Edwards Lifesciences, Medtronic Japan, Boston Scientific, Abbott Medical Japan, and the Daiichi-Sankyo Company. The sponsors were not involved in the study, data collection, statistical analyses, or manuscript writing. Dr Yamaguchi is a clinical proctor of transcatheter edge-to-edge repair at Abbott Medical; and has received a lecture fee and a scholarship donation from Abbott Medical. Drs. Kubo, Saji, Izumo, Watanabe, Asami, Yamamoto, Nakajima, Amaki, Ohno Enta, Shirai, Mizuno, Naganuma, Bota, Ueno, Mizutani, and Hayashida are clinical proctors of transcatheter edge-to-edge repair at Abbott Medical. Drs. Kubo, Saji, Izumo, Watanabe, and Amaki have received consultant fees from Abbott Medical. Drs. Asami, Yamamoto, and Nakajima have received lecture fees from Abbott Medical. Dr Kodama has received speaker fees from Abbott Medical. Dr Ohno has received consultant, advisor, and speaker fees from Abbott Medical. All other authors have reported that they have no relationships relevant to the contents of this paper to disclose.

## References

[1] Nkomo V.T., Gardin J.M., Skelton T.N., Gottdiener J.S., Scott C.G., Enriquez-Sarano M. (2006). Burden of valvular heart diseases: a population-based study. Lancet.

[2] Benjamin E.J., Virani S.S., Callaway C.W. (2018). Heart disease and stroke statistics-2018 update: a report from the American Heart Association. Circulation.

[3] Yeo I., Kim L.K., Wong S.C. (2019). Relation of hospital volume with in-hospital and 90-day outcomes after transcatheter mitral valve repair using MitraClip. Am J Cardiol.

[4] Nishimura R.A., Otto C.M., Bonow R.O. (2017). 2017 AHA/ACC focused update of the 2014 AHA/ACC Guideline for the management of patients with valvular heart disease: a report of the American College of Cardiology/American Heart Association Task Force on Clinical Practice Guidelines. J Am Coll Cardiol.

[5] Calafiore A.M., Iaco A.L., Tash A., Abukudair W., Di Mauro M. (2010). Mitral valve surgery for functional mitral regurgitation in patients with chronic heart failure—update of the results. Thorac Cardiovasc Surg.

[6] Goel S.S., Bajaj N., Aggarwal B. (2014). Prevalence and outcomes of unoperated patients with severe symptomatic mitral regurgitation and heart failure: comprehensive analysis to determine the potential role of MitraClip for this unmet need. J Am Coll Cardiol.

[7] Feldman T., Foster E., Glower D.D. (2011). Percutaneous repair or surgery for mitral regurgitation. N Engl J Med.

[8] Whitlow P.L., Feldman T., Pedersen W.R. (2012). Acute and 12-month results with catheter-based mitral valve leaflet repair: the EVEREST II (Endovascular Valve Edge-to-Edge Repair) High Risk Study. J Am Coll Cardiol.

[9] Stone G.W., Abraham W.T., Lindenfeld J. (2023). Five-year follow-up after transcatheter repair of secondary mitral regurgitation. N Engl J Med.

[10] Jokinen J.J., Hippelainen M.J., Pitkanen O.A., Hartikainen J.E. (2007). Mitral valve replacement versus repair: propensity-adjusted survival and quality-of-life analysis. Ann Thorac Surg.

[11] Bossone E., Di Benedetto G., Frigiola A. (2007). Valve surgery in octogenarians: in-hospital and long-term outcomes. Can J Cardiol.

[12] Kahn M.R., Robbins M.J., Kim M.C., Fuster V. (2013). Management of cardiovascular disease in patients with kidney disease. Nat Rev Cardiol.

[13] Samad Z., Sivak J.A., Phelan M., Schulte P.J., Patel U., Velazquez E.J. (2017). Prevalence and outcomes of left-sided valvular heart disease associated with chronic kidney disease. J Am Heart Assoc.

[14] Raheja H., Ahuja K.R., Nazir S. (2021). Association of baseline kidney disease with outcomes of transcatheter mitral valve repair by MitraClip. Catheter Cardiovasc Interv.

[15] Khan M.Z., Zahid S., Khan M.U. (2021). In-hospital outcomes of transcatheter mitral valve repair in patients with and without end stage renal disease: a national propensity match study. Catheter Cardiovasc Interv.

[16] Kubo S., Yamamoto M., Saji M. (2023). One-year outcomes and their relationship to residual mitral regurgitation after transcatheter edge-to-edge repair with MitraClip device: Insights from the OCEAN-Mitral registry. J Am Heart Assoc.

[17] Saji M., Yamamoto M., Kubo S. (2023). Short-term outcomes following transcatheter edge-to-edge repair: Insights from the OCEAN-Mitral registry. JACC Asia.

[18] Levey A.S., Coresh J., Balk E. (2003). National Kidney Foundation practice guidelines for chronic kidney disease: evaluation, classification, and stratification. Ann Intern Med.

[19] Ohno Y., Attizzani G.F., Capodanno D. (2016). Impact of chronic kidney disease on outcomes after percutaneous mitral valve repair with the MitraClip system: insights from the GRASP registry. EuroIntervention.

[20] Shah B., Villablanca P.A., Vemulapalli S. (2019). Outcomes after transcatheter mitral valve repair in patients with renal disease. Circ Cardiovasc Interv.

[21] Chhatriwalla A.K., Vemulapalli S., Holmes D.R. (2019). Institutional experience with transcatheter mitral valve repair and clinical outcomes: insights from the TVT registry. JACC Cardiovasc Interv.

[22] Sisinni A., Munafo A., Pivato C.A. (2022). Effect of chronic kidney disease on 5-year outcome in patients with heart failure and secondary mitral regurgitation undergoing percutaneous MitraClip insertion. Am J Cardiol.

[23] Matsumoto T., Kubo S., Izumo M., Mizuno S., Shirai S., MitraClip Japan P.M.S.I. (2022). MitraClip treatment of moderate-to-severe and severe mitral regurgitation in high surgical risk patients - Real-world 1-year outcomes from Japan. Circ J.

[24] Kar S., von Bardeleben R.S., Rottbauer W. (2023). Contemporary outcomes following transcatheter edge-to-edge repair: 1-year results from the EXPAND study. JACC Cardiovasc Interv.

[25] Puls M., Lubos E., Boekstegers P. (2016). One-year outcomes and predictors of mortality after MitraClip therapy in contemporary clinical practice: results from the German transcatheter mitral valve interventions registry. Eur Heart J.

[26] Sorajja P., Vemulapalli S., Feldman T. (2017). Outcomes with transcatheter mitral valve repair in the United States: an STS/ACC TVT Registry Report. J Am Coll Cardiol.

[27] Feldman T., Kar S., Rinaldi M. (2009). Percutaneous mitral repair with the MitraClip system: safety and midterm durability in the initial EVEREST (Endovascular Valve Edge-to-Edge REpair Study) cohort. J Am Coll Cardiol.

[28] Alon D., Stein G.Y., Korenfeld R., Fuchs S. (2013). Predictors and outcomes of infection-related hospital admissions of heart failure patients. PLoS One.

[29] Ueda T., Kawakami R., Horii M. (2014). Noncardiovascular death, especially infection, is a significant cause of death in elderly patients with acutely decompensated heart failure. J Card Fail.

[30] Drozd M., Garland E., Walker A.M.N. (2020). Infection-related hospitalization in heart failure with reduced ejection fraction: A prospective observational cohort study. Circ Heart Fail.

[31] Sarnak M.J., Levey A.S., Schoolwerth A.C. (2003). Kidney disease as a risk factor for development of cardiovascular disease: a statement from the American Heart Association Councils on Kidney in Cardiovascular Disease, High Blood Pressure Research, Clinical Cardiology, and Epidemiology and Prevention. Circulation.

[32] Tanaka K., Jujo K., Yamaguchi J., Ogawa H., Hagiwara N. (2019). Optimal blood pressure in patients with coronary artery disease and chronic kidney disease: HIJ-CREATE Substudy. Am J Med Sci.

[33] Shah B., Villablanca P.A., Vemulapalli S. (2019). Outcomes after transcatheter mitral valve repair in patients with renal disease. Cardiovasc Interv.

[34] Chiarito M., Pagnesi M., Martino E.A. (2018). Outcome after percutaneous edge-to-edge mitral repair for functional and degenerative mitral regurgitation: a systematic review and meta-analysis. Heart.

