# Supplementary material for: Effect of Renal Impairment on Clinical Outcomes After Mitral Valve Transcatheter Edge-to-Edge Repair
Source: JACC Asia. 2025 Jan 14;5(2):273–82. doi: 10.1016/j.jacasi.2024.10.025 (PMC11840231; doi:10.1016/j.jacasi.2024.10.025)

**Effect of Renal Impairment on Clinical Outcomes After Mitral Valve Transcatheter Edge‐to‐Edge Repair**

**Running title:** Impact of baseline renal impairment after M-TEER

Kazuki Tanaka ^a^, MD, Junichi Yamaguchi ^a^, MD, Masafumi Yoshikawa ^a^, MD, Eiji Shibahashi ^a^, MD, Hisao Otsuki ^a^, MD, Takanori Kawamoto ^a^, MD, Chihiro Koyanagi^a^, MD, Yusuke Inagaki ^a^, MD, Tomohito Kogure ^a^, MD, Masanori Yamamoto ^b,c,d^, MD, Mike Saji ^e,f^, MD, Masahiko Asami ^g^, MD,　Masaki Nakashima ^h^ Yusuke Enta ^h^, Shinichi Shirai ^i^, MD, Masaki Izumo ^j^, MD, Shingo Mizuno ^k^, MD, Yusuke Watanabe ^l^, MD, Makoto Amaki ^m^, MD, Kazuhisa Kodama ^n^, MD, Shunsuke Kubo ^o^, MD, Yoshifumi Nakajima ^p^, MD, Toru Naganuma ^q^, MD, Hiroki Bota ^r^, MD, Yohei Ohno ^s^, MD, Masahiro Yamawaki ^t^, MD, Hiroshi Ueno ^u^, MD, Kazuki Mizutani ^v^, MD, Toshiaki Otsuka ^w^, MD, Kentaro Hayashida ^x^, MD; OCEAN-Mitral investigators

**Supplemental Table 1: Cause of all-cause death**

**Supplemental Figure 1: Analysis of missing data of the data set for the present analysis.**

**Supplemental Figure 2A: Kaplan–Meier analysis for all-cause death in functional MR group.**

MR, mitral regurgitation

**Supplemental Figure 2B: Kaplan–Meier analysis for all-cause death in the degenerative MR group.**

MR, mitral regurgitation

**Supplemental Figure 2C: Kaplan–Meier analysis for HF hospitalization in degenerative MR group.**

HF, heart failure; MR, mitral regurgitation

**Supplemental Figure 2D: Kaplan–Meier analysis for HF hospitalization in degenerative MR group.**

HF, heart failure; MR, mitral regurgitation

**Supplemental Table 1. Cause of all-cause death**

|  | Normal eGFR group | Renal impairment group | Dialysis group |
| --- | --- | --- | --- |
|  | (n=291) | (n=1752) | (n=107) |
| All-cause death | 37 (12.7) | 351 (20.0) | 40 (37.4) |
| Cardiovascular death | 22 (7.6) | 222 (12.7) | 18 (16.8) |
| Non-cardiovascular death | 15 (5.2) | 129 (7.4) | 22 (20.6) |
| Infection | 4 (1.4) | 51 (2.9) | 14 (13.1) |
| Malignancy | 2 (0.7) | 24 (1.4) | 2 (1.9) |
| Stroke | 3 (1.0) | 4 (0.2) | 0 (0.0) |
| Gastrointestinal Bleeding | 0 (0.0) | 4 (0.2) | 0 (0.0) |
| Others | 5 (1.7) | 31 (1.8) | 4 (3.7) |

**Supplemental Figure 1:** Analysis of missing data of the data set for the present analysis.

**
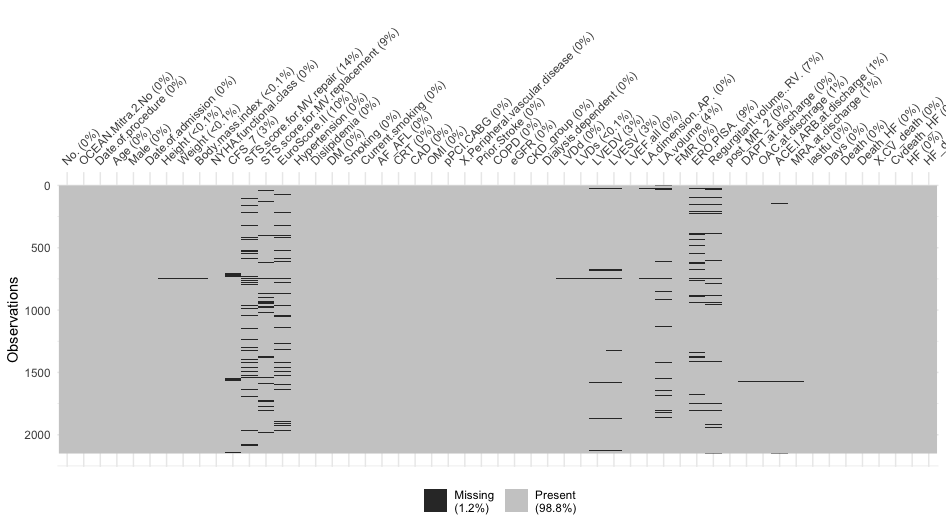
**

**Supplemental Figure 2A: Kaplan–Meier analysis for all-cause death in functional MR group.**


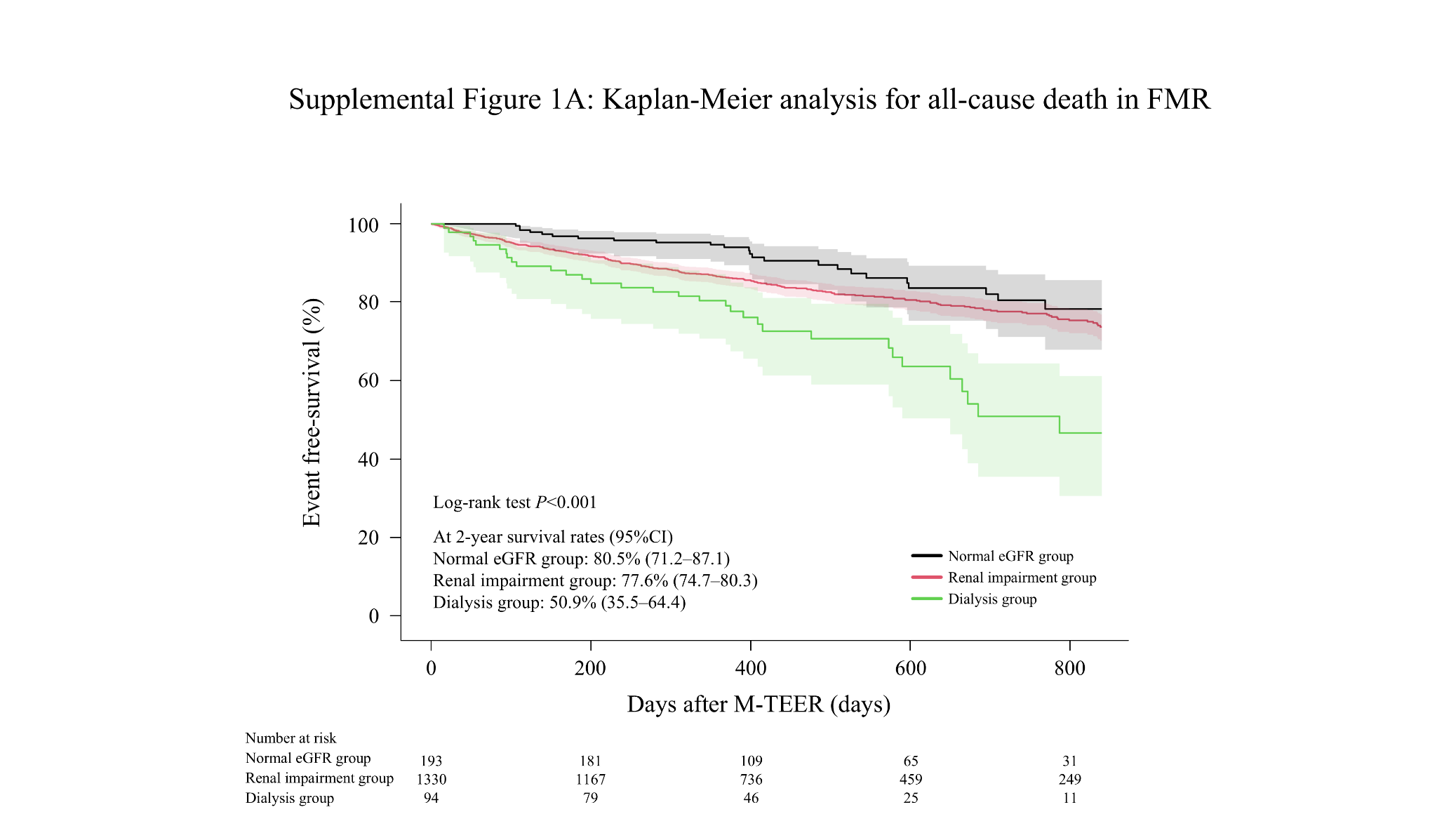


**Supplemental Figure 2B: Kaplan–Meier analysis for all-cause death in the degenerative MR group.**


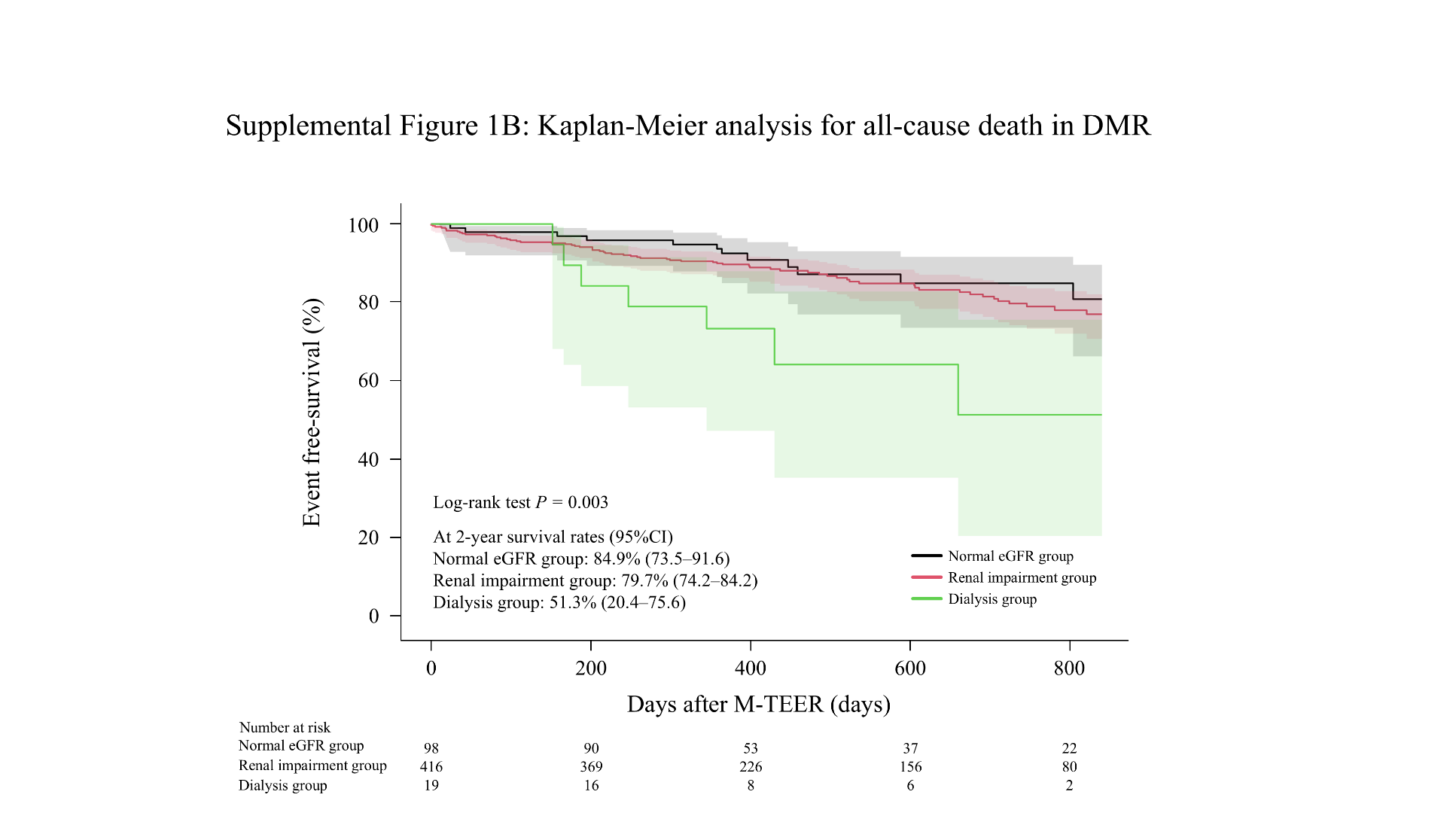


**Supplemental Figure 2C: Kaplan–Meier analysis for HF hospitalization in degenerative MR group.**


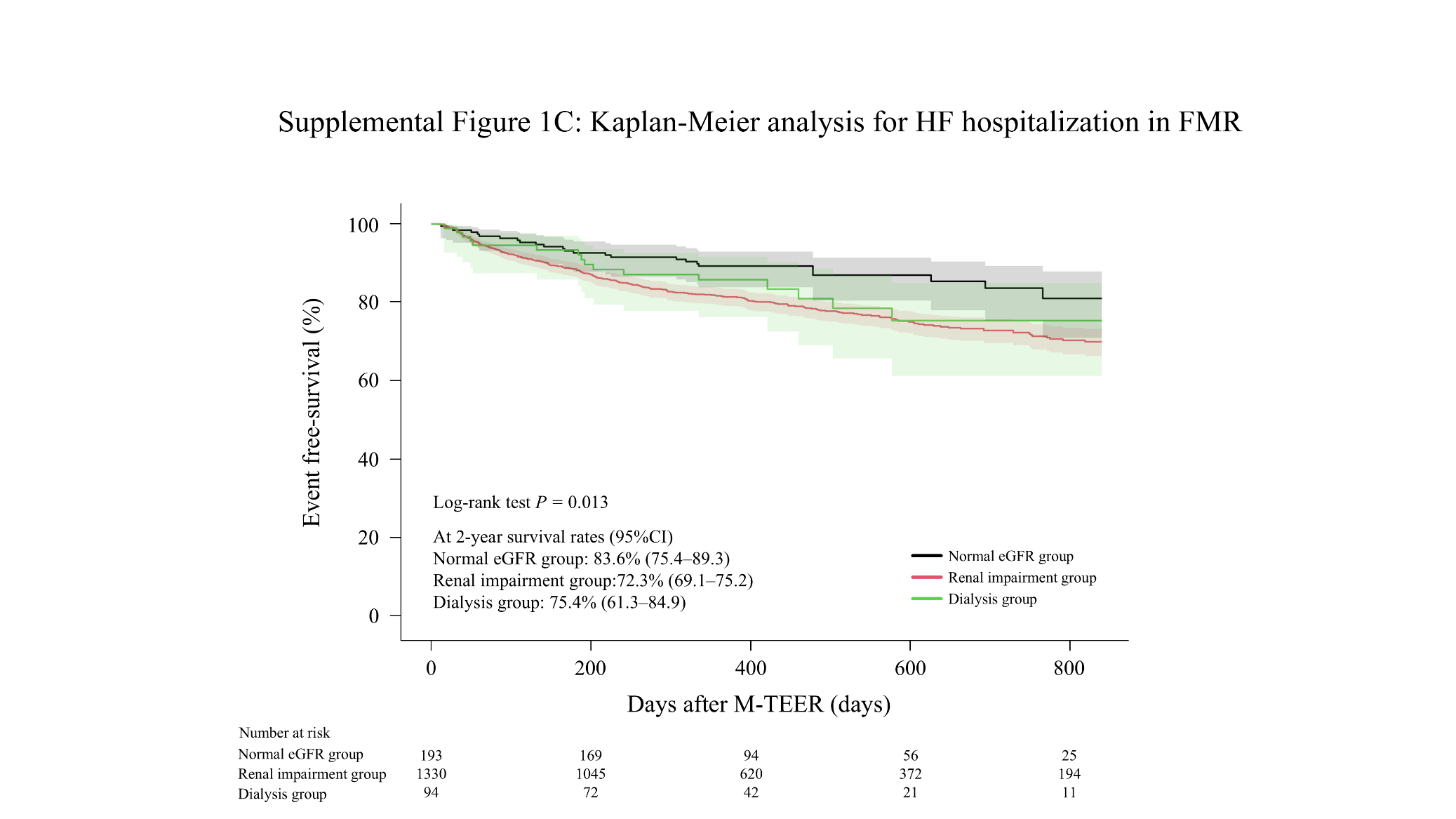


**Supplemental Figure 2D: Kaplan–Meier analysis for HF hospitalization in degenerative MR group.**


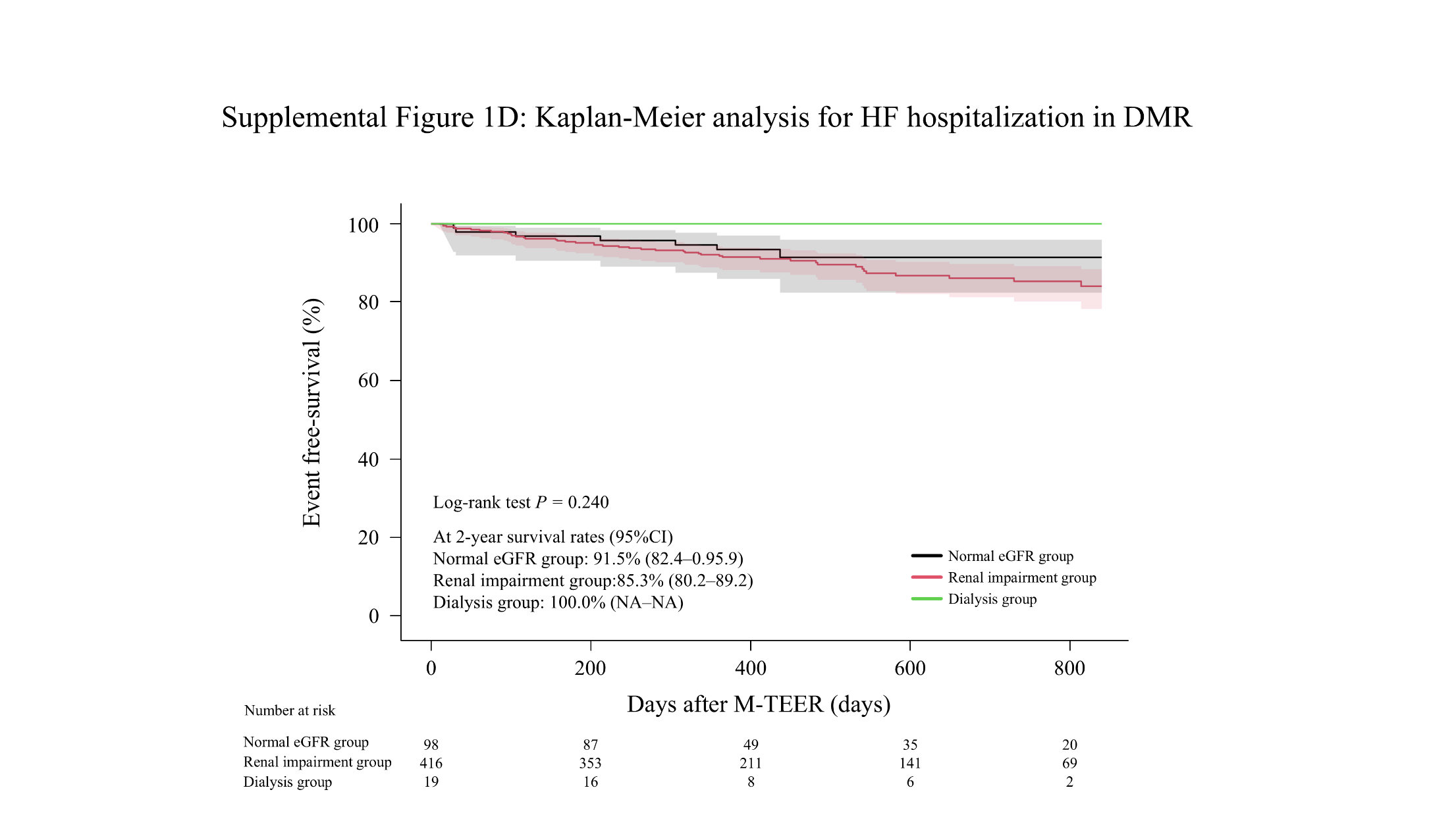

Supplement: Supplemental Material [file mmc1.docx]
